# Evaluation of cerebrospinal fluid glycoprotein NMB (GPNMB) as a potential biomarker for Alzheimer’s disease

**DOI:** 10.1186/s13195-021-00828-1

**Published:** 2021-05-04

**Authors:** Freyja Aichholzer, Hans-Wolfgang Klafki, Isabella Ogorek, Jonathan Vogelgsang, Jens Wiltfang, Norbert Scherbaum, Sascha Weggen, Oliver Wirths

**Affiliations:** 1 Department of Psychiatry and Psychotherapy, University Medical Center (UMG), Georg-August-University, Von-Siebold-Str. 5, 37075 Göttingen, Germany; 2 Department of Neuropathology, Heinrich-Heine-University, Düsseldorf, Germany; 3 Neurosciences and Signaling Group, Department of Medical Sciences, Institute of Biomedicine (ibiMED), University of Aveiro, Aveiro, Portugal; 4 German Center for Neurodegenerative Diseases (DZNE), Göttingen, Germany; 5 LVR-Hospital Essen, Department of Psychiatry and Psychotherapy, Medical Faculty, University of Duisburg-Essen, Essen, Germany

**Keywords:** Alzheimer’s disease, GPNMB, Cerebrospinal fluid, Biomarker, Inflammation, Immunoassay

## Abstract

**Background:**

Alzheimer’s disease (AD) is a neurodegenerative disorder associated with extracellular amyloid-β peptide deposition and progressive neuron loss. Strong evidence supports that neuroinflammatory changes such as the activation of astrocytes and microglia cells are important in the disease process. Glycoprotein nonmetastatic melanoma protein B (GPNMB) is a transmembrane glycoprotein that has recently been associated with an emerging role in neuroinflammation, which has been reported to be increased in post-mortem brain samples from AD and Parkinson’s disease patients.

**Methods:**

The present study describes the partial “fit for purpose” validation of a commercially available immunoassay for the determination of GPNMB levels in the cerebrospinal fluid (CSF). We further assessed the applicability of GPNMB as a potential biomarker for AD in two different cohorts that were defined by biomarker-supported clinical diagnosis or by neuroimaging with amyloid positron emission tomography, respectively.

**Results:**

The results indicated that CSF GPNMB levels could not distinguish between AD or controls with other neurological diseases but correlated with other parameters such as aging and CSF pTau levels.

**Conclusions:**

The findings of this study do not support GPNMB in CSF as a valuable neurochemical diagnostic biomarker of AD but warrant further studies employing healthy control individuals.

**Supplementary Information:**

The online version contains supplementary material available at 10.1186/s13195-021-00828-1.

## Introduction

Alzheimer’s disease (AD) is a common neurodegenerative disorder with a high demand for care that is putting an enormous strain on global healthcare systems. A substantial increase in the overall number of AD cases is expected due to demographic trends as age is the most important known non-genetic risk factor for the disease [1]. The two neuropathological hallmark lesions comprise extracellular amyloid-β (Aβ) plaques and intraneuronal neurofibrillary tangles (NFTs) [2]. Plaques are composed of Aβ peptides, which are to a large extent 40 or 42 amino acid long peptide fragments derived from the large transmembrane amyloid precursor protein (APP) through consecutive proteolytic cleavage events [3]. In contrast, NFTs are intracellular lesions composed of fibrillar aggregates of hyperphosphorylated microtubule-associated protein Tau. Tau aggregates are also abundant in other neurodegenerative diseases such as frontotemporal dementia or Picks’ disease [4]. To facilitate and improve the early clinical diagnosis of AD, various neurochemical biomarkers in body fluids such as plasma or cerebrospinal fluid (CSF) are actively investigated [5]. In the CSF, low concentrations of Aβ42 as well as a reduced Aβ_42_/Aβ_40_ ratio in combination with increased levels of total Tau (tTau) and phosphorylated Tau (pTau) proteins represent accepted biomarkers supporting the diagnosis of AD dementia [6–8]. In recent years, neuroimaging tools for in vivo amyloid detection by positron emission tomography (PET) using tracers such as ^11^C-Pittburgh compound B, ^18^F-florbetaben, or ^18^F-flutemetamol became available, showing good inverse correlations with CSF Aβ42 levels [9–11]. Neuroinflammatory changes in the form of abundant micro- and astrogliosis are also invariant neuropathological features of neurodegenerative disorders [12]. Recent human genetics data suggest an important contribution of the innate immune system to AD pathogenesis [13–15]. For example, missense mutations in the triggering receptor expressed on myeloid cells 2 (TREM2) are associated with an increased AD risk [14, 16]. Mutations in TREM2 and in its binding partner TYROBP are also associated with Nasu-Hakola disease, a rare autosomal recessive disorder that is characterized by the formation of multifocal bone cysts and progressive presenile dementia [17, 18].

Recently, we reported that glycoprotein non-metastatic melanoma protein B (GPNMB) was strongly upregulated in an AD mouse model [19] and described GPNMB as a novel AD-associated marker that is expressed in a subset of activated microglia cells [20]. GPNMB (also designated as osteoactivin) is a type I transmembrane protein originally discovered in a melanoma cell line [21]. GPNMB appears to be associated with negative regulation of inflammatory processes and has been demonstrated to reduce pro-inflammatory cytokine secretion in macrophages [22]. Furthermore, GPNMB has been proposed to play a role in neuroinflammation [23], and a recent immunohistochemical study confirmed its localization in microglia in brains of patients suffering from AD or Nasu-Hakola disease [24]. Aside from its microglial localization, GPNMB shares other similarities with TREM2, which represents a potential biomarker for microglia activity in AD [25], as both proteins were shown to undergo ectodomain shedding by the protease ADAM10 [26, 27].

In a small pilot study, we previously reported elevated GPNMB levels in both brain tissue and CSF samples of sporadic AD patients [20]. Here, we evaluated soluble GPNMB levels in the CSF as a potential diagnostic biomarker of AD in two independent clinical samples. Notably, in one of the cohorts, the subjects were classified according to a biomarker-supported clinical diagnosis, while in the second sample, the dichotomization was based on amyloid positron emission tomography (PET) data.

## Material and methods

### CRISPR/Cas9-mediated GPNMB gene knockout in the human THP-1 monocytic cell line

Two independent guide RNA sequences were designed to target exon 2 of the human GPNMB gene using the online CRISPR design tool available at https://design.synthego.com/#/. The sequences of the two oligonucleotide pairs were as follows: 5′-CAC CGT GCT CCC TCA TGT AAG CAG A-3′ and 5′-AAA CTC TGC TTA CAT GAG GGA GCA C-3′ (Exon 2.1); 5′-CAC CGA AAG ACC TTC TGC TTA CAT G-3′ and 5′-AA AC CAT GTA AGC AGA AGG TCT TTC-3′ (Exon 2.2). A guide RNA targeting enhanced green fluorescent protein (EGFP) was used as a control (5′-CAC CGG GTG AAC CGC ATC GAG CTG A-3′ and 5′-AAA CTC AGC TCG ATG CGG TTC ACC C-3′) [28]. The oligonucleotide pairs were annealed, phosphorylated with polynucleotide kinase**,** and cloned into the lentiviral vector lentiCRISPRv2 (a gift from Feng Zhang, Addgene Plasmid #52961). This lentiviral one-vector system encodes *S. pyogenes* Cas9, a scaffold for cloning of a single guide RNA, and a puromycin resistance gene for stable selection [29]. Lentiviral particles were produced in 293FT cells using a third-generation lentivirus packaging system as described [30]. The human monocytic cell line THP-1 growing in solution was infected with the lentiviral particles for 24 h and stable mass cultures were selected with 0.3 μg/ml puromycin. Subsequently, limited dilution cloning was performed to obtain single cell clones. Individual cell clones were isolated and analyzed for target gene expression by qPCR.

### RNA extraction and quantitative PCR

For GPNMB expression analysis, THP-1 GPNMB knockout and control cells were seeded in 12-well plates in the presence of 10 nM phorbol myristate acetate (PMA), to induce differentiation of THP-1 monocytes into adherent macrophages. Forty-eight hours after seeding, total RNA extraction (ReliaPrep™ RNA Cell Miniprep System, Promega) and cDNA synthesis (using M-MLV (H-) reverse transcriptase, Promega) were performed according to the manufacturer’s instructions. For subsequent quantitative PCR analysis, the Platinum™ qPCR Super Mix (Thermo Fisher) based on the fluorescent nucleic acid dye SYBR™ Green was used. Ten microliters of the SYBR™ Green Mix, 500 nM of each primer, and 0.5 μl ROX™ reference dye were mixed, and RNase free water was added to a final volume of 17 μl. The reaction mix and 3 μl of the diluted cDNA were transferred into a 96-well reaction plate (Applied Biosystems). Quantitative PCR was performed in a StepOnePlus™ Real-Time PCR System (Applied Biosystems) using the following PCR program: 10 min for 95 °C followed by 40 cycles of repeated denaturation at 95 °C for 15 s and hybridization/elongation at 60 °C for 1 min. Relative gene expression was calculated using the ΔΔC_T_-method, with human ARF as a housekeeping gene. Primer sequences were as follows: GPNMB fwd 5′-TGC GGT GAA CCT GAT ATT CCC-3′ and rev 5′-CAG GGA AGA CGT TAT GAT GGC T-3′; ARF fwd 5′-GAC CAC GAT CCT CTA CAA GC-3′ and rev 5′-TCC CAC ACA GTG AAG CTG ATG-3′.

### THP-1 cell culture supernatant collection and lysate preparation

THP-1 GPNMB knockout and control cells were seeded in 6-well plates at a density of 700,000 cells/well in 2 ml complete growth medium (RPMI 1640 medium supplemented with 10% fetal calf serum, 1 mM sodium pyruvate, 5 units/ml penicillin, 5 μg/ml streptomycin, 2 mM L-glutamine, and 50 μM 2-ß-mercaptoethanol) in the presence of 10 nM PMA to induce differentiation in adherent macrophages. After 48 h, conditioned supernatants were collected, centrifuged at 18,000×*g* for 3 min to remove cell debris, and protease inhibitors (cOmplete, Merck) were added prior to storage at − 20 °C. Cells were washed twice with 1 ml PBS and lysed in NP40 buffer with added protease inhibitors. Protein concentrations of cell lysates were determined photometrically with a bicinchoninic acid protein assay kit (Pierce).

### Study cohort and collection procedures

CSF samples were retrieved from the local biobanks of the LVR-Hospital Essen, Department of Psychiatry and Psychotherapy, University of Duisburg-Essen (cohort 1), and the Department of Psychiatry and Psychotherapy, University Medical Center Goettingen (cohort 2). The respective local ethics committees approved the use of the archived samples for biomarker studies. Participants of cohort 1 were recruited at the Department of Psychiatry and Psychotherapy at the LVR-Hospital Essen, University of Duisburg-Essen, as well as in the Memory Clinic at the Elisabeth Hospital Essen (Germany). Participants of cohort 2 were recruited at the Department of Psychiatry and Psychotherapy at University Medical Center Goettingen. CSF samples were obtained by lumbar puncture for diagnostic reasons with informed consent from all subjects or their legal caregivers. CSF samples were collected in polypropylene tubes and centrifuged for 15 min at 1600×*g* at room temperature. The resulting supernatant (“CSF”) was stored in aliquots at − 80 °C until use, and aliquots were only thawed once prior to the analysis [31].

Study cohort 1 comprised a subgroup of the clinical sample previously reported and described in detail in Ref. [31] (Table 1). More specifically, cohort 1 included subjects categorized into the two diagnostic groups (i) probable AD (*n* = 54) and (ii) non-AD disease controls (DC) (*n* = 72). The DC group contained either non-demented patients comprising a variety of psychiatric or neurological disorders such as schizophrenia, normal pressure hydrocephalus, depressive disorders or addictive disorders and concomitant disease, as well as cases with dementia of other origin (such as vascular or frontotemporal dementia). The classification was based on a biomarker-supported clinical diagnosis, which considered NINCDS-ADRDA criteria and the CSF levels of total Tau, phospho-Tau 181 (pTau181), Aβ_1–42_, the Aβ_1–42/1–40_ ratio and additional CSF Aβ data obtained with the Meso Scale Discovery (MSD) V-Plex Aβ panel (6E10) multiplex assay (for details see Ref. [31]). In the current study, we included published MSD-multiplex CSF Aβ data from Ref. [31] in the statistical analysis without correction for age and center effects.

**Table 1:** Characteristics of study cohort 1 and baseline statistics of CSF measurements

Cohort 1			
	DC (***n*** = 72)	AD (***n*** = 54)	***p***-value AD - DC
**Age**	69.71 ± 10.68	72.76 ± 10.91	0.1184*
**Gender**			0.0057^$^
**Women**	35 (48.6%)	40 (74.1%)	
**Men**	37 (51.2%)	14 (25.9%)	
**CSF GPNMB [pg/ml]**	9806 ± 4006	11270 ± 4957	0.0788^#^
**APOE genotype**			
**ε2/ε2**	–	1	
**ε2/ε3**	10	4	
**ε2/ε4**	2	3	
**ε3/ε3**	44	12	
**ε3/ε4**	15	29	
**ε4/ε4**	1	5	
** ≥ 1** ***APOE*** **ε4 allele, n (%)**	18 (25%)	37 (68.5%)	< 0.0001^$^
CSF p-Tau [pg/ml]*	47.47 ± 18.89	111.5 ± 46.66	< 0.0001^#^
CSF t-Tau [pg/ml]*	257.1 ± 91.43	760.2 ± 310.2^§^	< 0.0001^#^
CSF Aβ38 [pg/ml]*	2271 ± 997.3	2543 ± 889.4	0.0902^#^
CSF Aβ40 [pg/ml]*	6871 ± 3050	7614 ± 2875	0.1843^#^
CSF Aβ42 [pg/ml]*	709.2 ± 394	359.7 ± 141.6	< 0.0001^#^
CSF Aβ 42/40*	0.0994 ± 0.0231	0.0478 ± 0.0104	< 0.0001*

The indicated parameters had been determined and partially reported before in a previous study [31]. ^§^Some samples (20%) exceeded the upper limit of detection of the assay and were set to 1200 pg/ml. *Unpaired *t*-test, ^#^Mann-Whitney test, or ^$^Fisher’s exact test for differences between the diagnostic groups

The subjects in study cohort 2 (Table 3) were dichotomized into the categories amyloid-PET-positive (PET^+^) and amyloid-PET-negative (PET^−^) according to the results of an amyloid-PET/CT examination using the tracers ^18^F-Florbetaben [32] or ^18^F-Florbetapir. For a subset of cases (*n* = 27) PET standard uptake value ratios (SUVRs) were also available. The CSF-concentrations of t-Tau and pTau181 were measured routinely in the laboratory of Clinical Chemistry, University Medical Center Goettingen. The CSF concentrations of Aβ_38_, Aβ_40_, and Aβ_42_ were determined in the context of this study with the V-Plex Aβ panel 1 (6E10) multiplex assay kit (MSD) after 16-fold dilution of the CSF samples.

### GPNMB analysis in cell culture supernatants and CSF

GPNMB was measured in conditioned cell culture supernatants, cell lysates, and diluted CSF with the human osteoactivin R-PLEX antibody set ((#F21ZH-3) (MSD, Gaithersburg, USA), with an antibody pair raised against the extracellular domain of recombinant osteoactivin (amino acids Lys23-Asn486), employing MSD GOLD 96-well Small Spot Streptavidin SECTOR plates (#L45SA-2) according to the manufacturer’s instructions.

Calibrator peptide dilutions were prepared in Diluent-7, while the detection antibody solution was prepared in Diluent-3 (MSD). Cell culture supernatants were measured after 1:2 dilution with Diluent-7, while cell lysates were measured after 20-fold dilution (3 technical replicates each).

CSF aliquots were thawed on ice and diluted 1:10 with Diluent-7 prior to the measurements. For diluting calibrator peptides and samples, Protein LoBind Tubes (Eppendorf AG, Germany) were used. For coating, the plates were incubated for 60 min at room temperature with continuous agitation with 25 μL per well of biotinylated capture antibody diluted in Diluent-100. After three washes with wash buffer, 150 μL per well of the calibrator peptide dilutions or diluted samples were added, and the plate was incubated for 60 min at room temperature with continuous agitation. After three washing steps, 150 μL per well of diluted detection antibody were added and incubated for 60 min. Finally, the plate was washed 3 times with wash buffer, followed by addition of 150 μl per well of MSD GOLD Read Buffer. Electrochemiluminescent signals were recorded on a MESO QuickPlex SQ 120 instrument (MSD) and analyzed with the Discovery Workbench software. For normalization purposes, duplicates of pooled normal human CSF (Lot number IPLA-CSFP-113017, Innovative Research) were included on all assay plates.

### APOE genotyping

*APOE* status was determined using a quantitative real-time PCR protocol as described previously [33]. All samples were measured in duplicates for all primer combinations including negative controls. Analyses were carried out using a CFX Connect Real-Time PCR system (BIO-RAD).

### Statistical analysis

All data have been tested for normally distributed variables with the Shapiro-Wilk test to ensure that parametric test can be applied. When parametric testing was possible, all group differences were calculated with unpaired *t*-test, Fisher’s exact test, or Mann-Whitney test in case of non-parametric testing. Statistical evaluations were done with GraphPad Prism 9.

## Results

### CRISPR/Cas9-mediated gene knockout in THP-1 cells

**Fig. 1:**
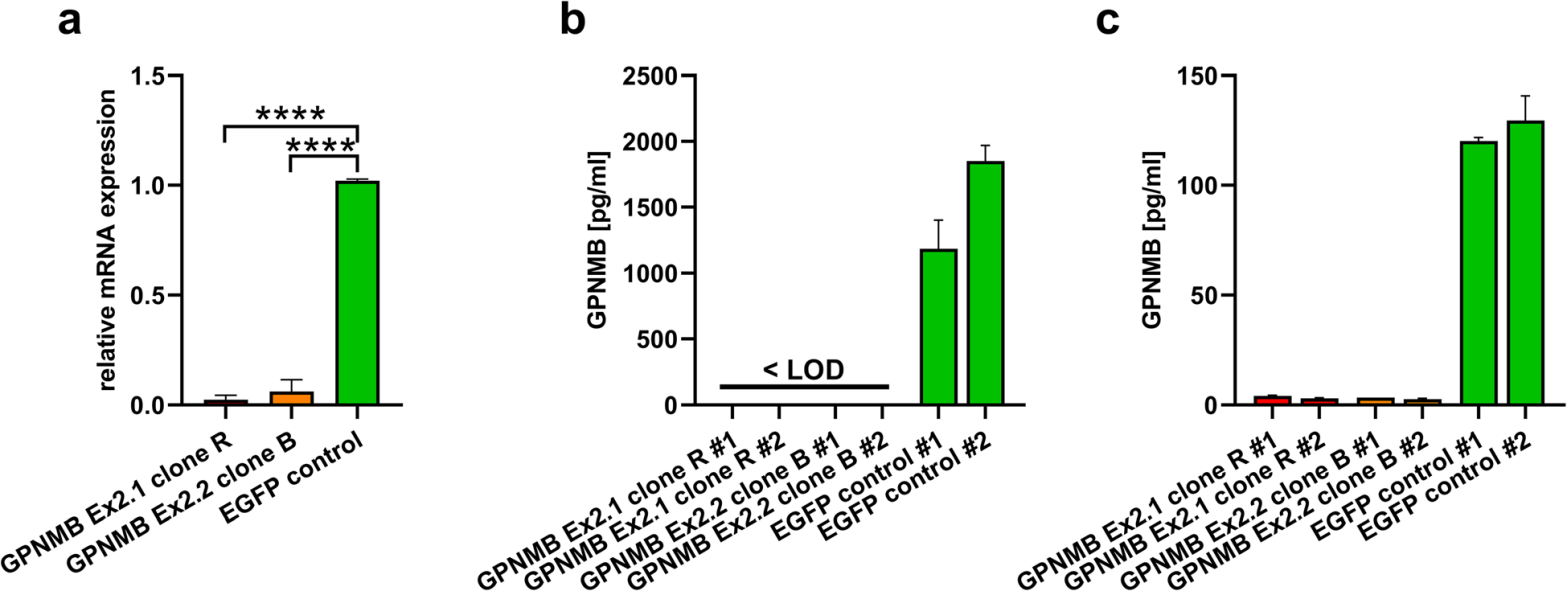
Targeted GPNMB-knock-out in THP-1 cells. RT-PCR analysis for GPNMB revealed significantly reduced GPNMB RNA expression levels in GPNMP knock-out THP-1 cell clones (Ex.2) **a** In comparison to EGFP-expressing control cells, GPNMB protein levels were significantly reduced or below level of detection (LOD) in **b** supernatants or **c** cell lysates from THP-1 GPNMB knock-out clones; ****p < 0.0001

Macrophage-like cells derived from the human monocytic cell line THP-1 have been demonstrated to express high levels of GPNMB [22]. Hence, to establish a cell-based control to investigate potential cross-reactivity of a commercial GPNMB immunoassay, GPNMB deficient THP-1 cells were generated by CRISPR/Cas9-mediated gene knockout. To confirm the successful GPNMB-knockout, THP-1 cells were differentiated to adherent macrophage-like cells by treatment with PMA, and RNA was extracted and reverse-transcribed into cDNA from control cells transduced with a guide RNA targeting enhanced green fluorescent protein (EGFP), as well as from two individual cell clones transduced with guide RNAs targeting two distinct sequences in exon 2 of the human GPNMB gene (GPNMB Ex2.1, clone R; GPNMB Ex2.2 clone B). In the GPNMB-targeted cell clones, PCR analysis confirmed a ~97% (GPNMB Ex2.1 clone R) and ~94% (GPNMB Ex2.2 clone B) reduced expression of human GPNMB compared to the EGFP-targeted control cells (both *p* <  0.0001; Fig. 1a).

Next, conditioned cell supernatants and cellular lysates were collected from THP-1 derived macrophages and GPNMB protein levels were measured with the osteoactivin R-PLEX assay. Supernatants derived from the EGFP-targeted control cells showed GPNMB levels of > 1000 pg/ml. In contrast, GPNMB signals in the supernatants of the GPNMB-targeted cells were below the detection level (Fig. 1b). This was also observed for intracellular GPNMB levels. While robust GPNMB concentrations of ~ 125 pg/ml were measured in cell lysates prepared from the EGFP control cells, the protein was barely detectable in the two GPNMB knockout cell clones (~ 3 pg/ml) (Fig. 1c). Taken together, these results provide evidence for high selectivity of the GPNMB protein detection assay.

### Impact of sample dilution on GPNMB measurement in CSF and inter-assay variance

A pooled control CSF sample and three randomly selected individual CSF samples were measured in duplicates either undiluted or after 2-, 5-, 10-, and 20-fold dilution. The CSF GPNMB concentrations were back-calculated and plotted against the dilution factor for each sample.

For all analyzed samples, the back-calculated CSF concentrations did not show substantial variation between the different dilutions, suggesting that the impact of interfering substances producing so-called matrix effects was negligible (see Additional file 1). Therefore, we selected a 10-fold dilution for all subsequent CSF measurements, representing a reasonable relationship between applied sample volumes and measuring accuracy. Using the Discovery Workbench software (MSD), the lower limit of detection (LLOD) was automatically determined and defined as the lowest concentration producing a detectable signal three standard deviations above the zero calibrator (“blank”) value. Accordingly, the lower limit of quantification (LLOQ) was calculated as the lowest concentration necessary to generate signals 10 standard deviations above the zero calibrator (blank) [31]. We included pooled control CSF in all assay plates as a quality control sample for normalization and to evaluate inter-assay variance. Within this study, two different assay lots were applied, showing between plate coefficients of variance (% CV) of 4.3% and 6.4% respectively.

### Study cohort 1: CSF GPNMB levels in probable AD patients and age-matched disease controls classified by a biomarker-supported clinical diagnosis

**Fig. 2:**
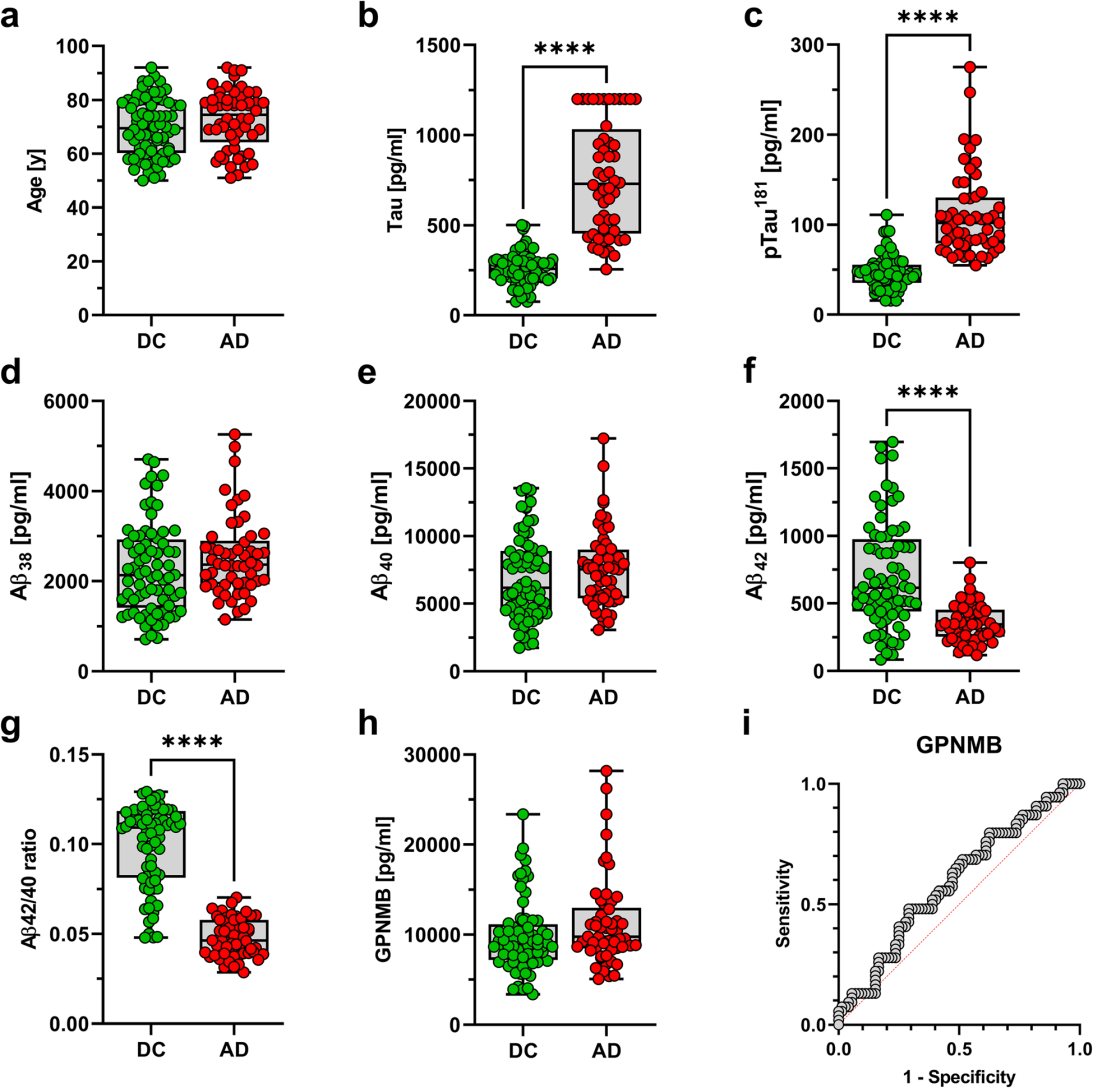
While DC and AD groups in study cohort 1 did not show a significant age difference (**a**), AD patients showed significantly elevated **b** total Tau and **c** pTau181 levels. An analysis of CSF Aβ peptides revealed unchanged **d** Aβ_38_ and **e** Aβ_40_ levels, but significantly reduced **f** Aβ_42_ and **g** Aβ_42/40_ ratios in the AD group. **h** CSF GPNMB levels were not significantly altered and a ROC analysis **i** with GPNMB CSF levels showed only poor discrimination between the groups (AUC = 0.59); ****p < 0.0001

The characteristics of study cohort 1 and baseline statistics of CSF biomarker data are summarized in Table 1. In 20% of the samples in the AD group, total Tau CSF levels exceeded the assay range and were set to 1200 pg/ml representing the upper limit of quantification of the assay. The histograms of the distribution of the CSF Aβ_42/40_ ratio and GPNMB levels are shown in Additional file 2. In line with previous results, a bimodal distribution of the CSF Aβ_42/40_ ratio was also observed in this sub-cohort of the formerly published data set [31]. There was no statistically significant difference with regard to age between the AD and the disease control (DC) control group (*p* = 0.1312, Table 1, Fig. 2a). The DC group comprised significantly less females (*p* <  0.01) and contained significantly fewer individuals carrying at least one *APOE* ε4 allele (*p* <  0.0001; Table 1). CSF total Tau and CSF pTau^181^ were elevated and CSF Aβ_42_ and the CSF Aβ_42/40_ ratio (measured with MSD assay) were reduced in the AD patients. This was expected, because CSF-biomarkers including Aβ had been considered for the biomarker-supported clinical diagnosis/classification (see [31]). The CSF levels of Aβ_40_ (MSD) and Aβ_38_ (MSD) did not show statistically significant differences between the diagnostic groups (Fig. 2b–g). The GPNMB CSF levels were not statistically significantly altered in AD patients compared to the DC group (*p* = 0.079; Fig. 2h).

### Associations of CSF GPNMB levels with CSF biomarkers

**Fig. 3:**
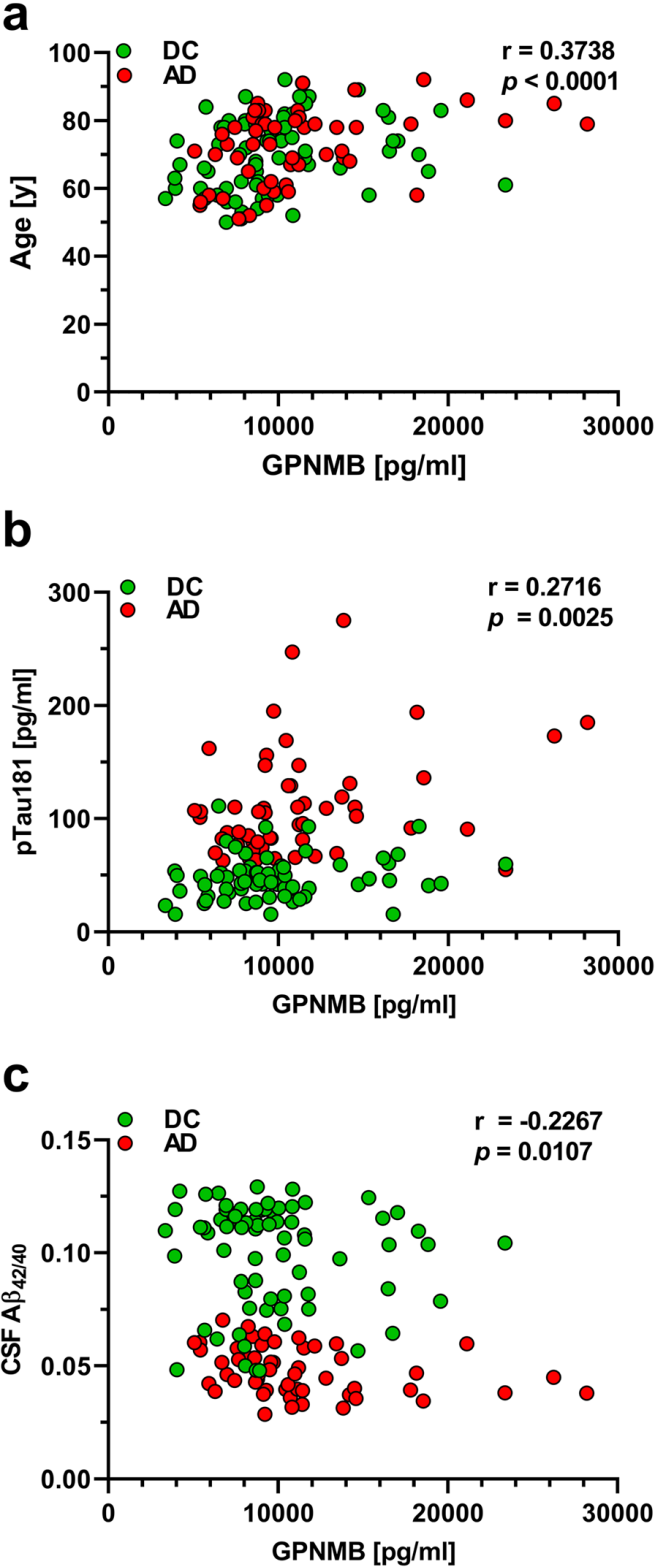
MSD GPNMB measurements in study cohort 1 were significantly correlated with **a** age, as well as **b** pTau181 and the **c** CSF Aβ_42/40_ ratio

In the entire sample, as well as in the separate AD and DC groups, the CSF GPNMB levels showed a significant positive correlation with age (Table 2 and Fig. 3a). In addition, pTau^181^ correlated significantly with GPNMB levels in the entire sample and the AD group (Table 2 and Fig. 3b). Neither CSF Aβ_38_, Aβ_40_, nor Aβ_42_ showed a correlation with CSF GPNMB levels. However, statistically significant inverse correlations of the CSF Aβ_42/40_ ratio and the GPNMB CSF levels were observed in the total sample and the AD group (Table 2, Fig. 3c).

**Table 2:** Analysis of correlations between CSF-GPNMB and other parameters in study cohort 1

Correlations	CSF GPNMB		
	Total sample	Controls	AD
**Age**	0.3738 (< 0.0001)	0.3970 (0.0004)	0.4225 (0.0015)
**CSF pTau181**	0.2716 (0.0025)	0.1834 (0.1315)	0.3114 (0.0232)
**MSD-CSF Aβ38**	0.1108 (0.2168)	0.0918 (0.4432)	0.0784 (0.5727)
**MSD-CSF Aβ40**	0.0860 (0.3381)	0.0894 (0.4552)	0.0359 (0.7964)
**MSD-CSF Aβ42**	−0.0771 (0.3907)	0.0619 (0.6053)	−0.1257 (0.3649)
**MSD-CSF Aβ42/40**	−0.2267 (0.0107)	−0.1003 (0.4020)	− 0.3888 (0.0049)

Spearman’s rho (*p*-value), *CSF* cerebrospinal fluid

The area under the receiver operating characteristics (ROC) curve (AUC) was 0.59 for CSF GPNMB alone (95% CI 0.49, 0.69) (Fig. 2i), indicating poor ability to discriminate between AD patients and dementia of other causes. The AUCs based on either age or APOE e4 status alone were 0.58 (95% CI 0.48, 0.68) and 0.72 (95% CI 0.63, 0.81) respectively. When we added GPNMB as the potential biomarker of interest to these models, no obvious change in the AUC combining age and GPNMB was noted (AUC 0.59; 95% CI 0.50, 0.70). However, combining GPNMB and *APOE* ε4 status resulted in a slightly increased AUC of 0.77 (95% CI 0.69, 0.85) (see Additional file 3).

From the ROC analysis, we calculated a maximum Youden index (Youden index J = sensitivity + specificity – 1) [34, 35] at a cutpoint of > 10,429 pg/ml for the CSF GPNMB concentration. At this optimum cut-point, CSF GPNMB levels had low sensitivity (48.15%) and specificity (70.83%) and correctly identified 25 of the 54 AD cases and 50 of the 72 controls. Twenty-two control patients (17.5%) were misclassified as AD (false positives) and 29 AD patients (23%) were misclassified as controls (false negatives) (see Additional file 3).

### Study cohort 2: CSF GPNMB levels in a clinical sample dichotomized according to amyloid PET analysis

**Fig. 4:**
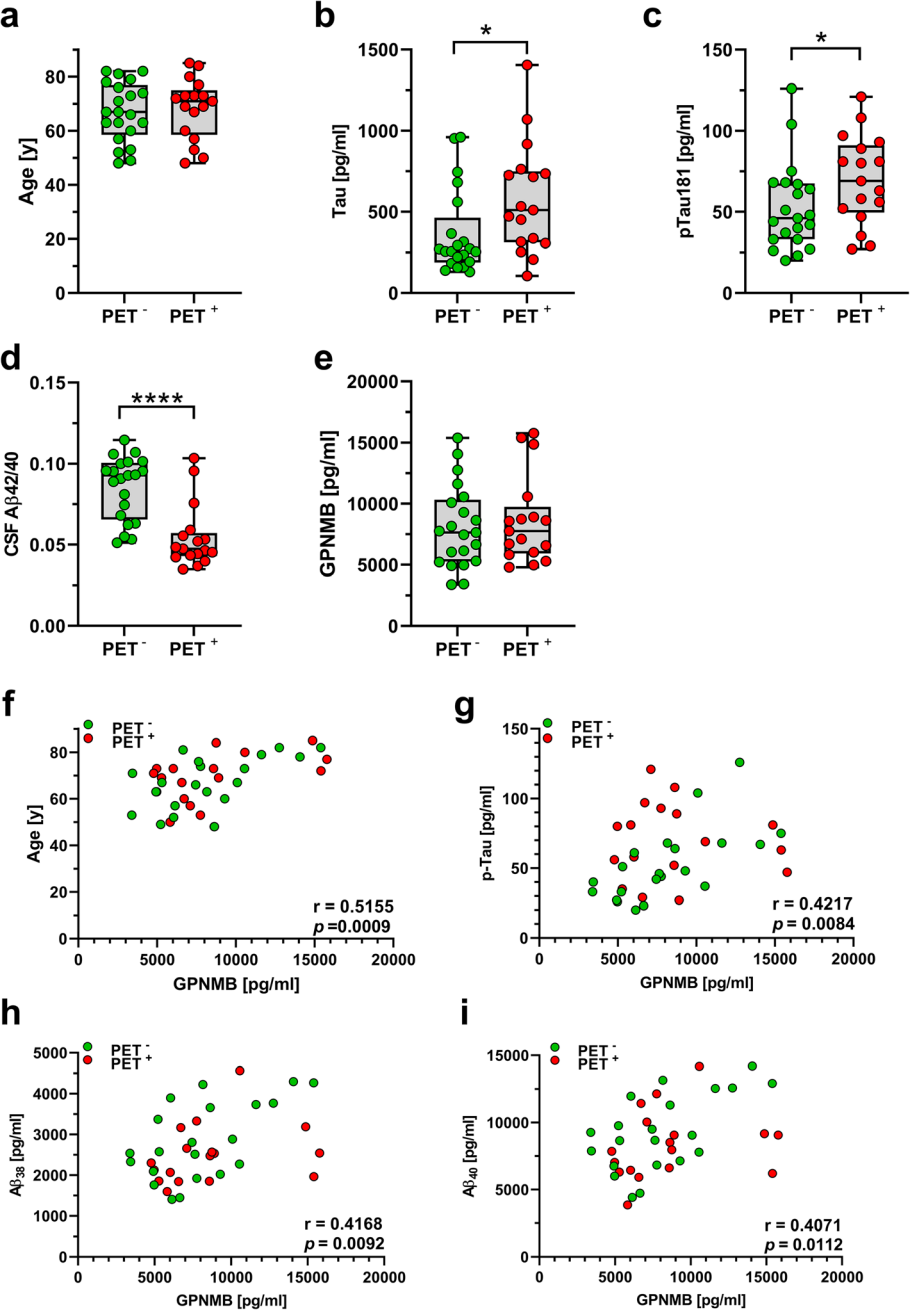
No significant difference was evident in the **a** age distribution among PET^+^ and PET^−^ individuals in study cohort 2. CSF levels of **b** total Tau and **c** pTau181 were increased in PET^+^ subjects, together with a significant decrease in the **d** CSF Aβ_42/40_ ratio, while **e** CSF GPNMB levels were not significantly altered. In this cohort, CSF GPNMB levels were significantly correlated with **f** age, **g** pTau181 (G), as well as **h** CSF Aβ_38_ (H) and **i** Aβ_40_; *p < 0.05, ****p < 0.0001

We have previously shown that GPNMB co-localizes with a distinct population of IBA1-positive microglia cells in the vicinity of Aβ plaques [20]. To assess whether soluble GPNMB levels in CSF might be associated with amyloid PET evidence of brain amyloid plaque pathology, 39 CSF samples from patients in the biomaterial bank of the Department of Psychiatry and Psychotherapy of the University Medical Center Göttingen were investigated. All individuals in this cohort underwent lumbar puncture for CSF biomarker analysis and amyloid-PET/CT. Thirty-eight samples were finally included in the statistical evaluation. One subject was identified as an outlier regarding several parameters by Grubbs test and was therefore excluded from the final analysis. The characteristics of study cohort 2 and baseline statistics of CSF biomarker data are summarized in Table 3. The histograms of the distribution of the CSF Aβ_42/40_ ratio and of GPNMB levels are shown in Additional file 4. No statistical differences regarding age, gender, or the presence of one or more APOE ε4 alleles were detected between PET^+^ and PET^−^ groups (Table 3, Fig. 4a). With regard to CSF biomarkers, PET^+^ individuals showed significantly increased total Tau and pTau^181^ levels (both *p* <  0.05), as well as a significantly decreased CSF Aβ_42/40_ ratio (*p* <  0.0001, Fig. 4b–d). The PET SUVR values were statistically significantly correlated with CSF Aβ_42_ levels (*r* = − 0.599; *p* = 0.0010) and the CSF Aβ_42/40_ ratio (*r* = − 0.658; *p* = 0.0002).

**Table 3:** Characteristics of study cohort 2 and baseline statistics of CSF measurements

Cohort 2			
	PET^**−**^ (***n*** = 21)	PET^**+**^ (***n*** = 17)	***p***-value PET^**−**^ - PET^**+#**^
**Age**	66.86 ± 10.95	68.29 ± 11.20	0.6928*
**Gender**			0.7442^$^
**Women**	9 (42.9%)	6 (35.3%)	
**Men**	12 (57.1%)	11 (64.7%)	
**CSF GPNMB [pg/ml]**	8074 ± 3358	8620 ± 3577	0.6845^#^
**APOE genotype**			
**ε2/ε2**	–	–	
**ε2/ε3**	2	–	
**ε2/ε4**	1	1	
**ε3/ε3**	13	7	
**ε3/ε4**	4	8	
**ε4/ε4**	1	1	
**≥ 1** ***APOE*** **ε4 allele, n (%)**	6 (28.6%)	10 (58.8%)	0.0990^$^
**CSF p-Tau [pg/ml]**	52.52 ± 26.59	69.76 ± 27.39	0.0367^#^
**CSF t-Tau [pg/ml]**	362.3 ± 258.4	578.0 ± 339.1	0.0225^#^
**CSF Aβ38 [pg/ml]**	2848 ± 946.1	2509 ± 734.8	0.2810^#^
**CSF Aβ40 [pg/ml]**	9294 ± 2851	8343 ± 2575	0.2929*
**vCSF Aβ42 [pg/ml]**	796.5 ± 336.9	441.2 ± 157.3	0.0002^#^
**CSF Aβ ratio 42/40**	0.08529 ± 0.01939	0.05433 ± 0.01949	< 0.0001^#^

*Unpaired *t*-test, ^#^Mann-Whitney, or ^$^Fisher’s exact test for differences between the diagnostic groups

The CSF GPNMB levels did not show a statistically significant difference between PET^+^ and PET^−^ individuals (*p* = 0.685, Fig. 4e).

### Correlation analyses between CSF GPNMB and other parameters

CSF GPNMB levels were significantly and positively correlated with age in the entire sample set and in the PET^−^ group, but not in the PET^+^ individuals (Table 4 and Fig. 4f). Total Tau levels in the CSF were not statistically significantly associated with GPNMB in any of the groups. However, significant correlations between GPNMB and pTau^181^ were observed in the total sample and in the PET^−^ patients, but not in the PET^+^ subgroup (Table 4 and Fig. 4g). CSF Aβ_38_ and Aβ_40_ levels were statistically significantly associated with GPNMB levels in the entire sample and in the PET^−^ group, but not in the PET^+^ group (Table 4 and Fig. 4h, i). Neither CSF Aβ_42_ levels nor the CSF Aβ_42/40_ ratio showed statistically significant correlations with CSF GPNMB levels in this sample set (Table 4).

**Table 4:** Analysis of correlations between CSF-GPNMB and other parameters in study cohort 2

Correlations	CSF GPNMB		
	Total sample	PET^**−**^	PET^**+**^
**Age**	*0.5155 (0.0009)*	*0.5872 (0.0051)*	*0.4280 (0.0866)*
**CSF total Tau**	0.3001 (0.0671)	0.4714 (0.0310)	0.0147 (0.9585)
**CSF pTau181**	0.4217 (0.0084)	0.7206 (0.0002)	0.0189 (0.9454)
**CSF Aβ38**	0.4168 (0.0092)	0.4935 (0.0230)	0.4461 (0.0744)
**CSF Aβ40**	*0.4071 (0.0112)*	*0.5971 (0.0043)*	*0.2144 (0.4086)*
**CSF Aβ42**	0.1581 (0.3431)	0.3831 (0.0865)	0.1642 (0.5276)
**CSF ratio Aβ42/40**	−0.1238 (0.4591)	−0.0273 (0.9066)	−0.0392 (0.8835)

*Pearson’s rho (p-value)*

Spearman’s rho (*p*-value)

*CSF* cerebrospinal fluid

The AUC for the CSF Aβ_42/40_ ratio measured with the MSD assay for the discrimination between PET^+^ and PET^−^ was 0.86 (95% CI 0.74, 0.99). The maximum Youden index calculated from the ROC analysis was 0.6806 at the cut-point < 0.06071 for the Aβ_42/40_ ratio. At this optimum cut-point, the CSF Aβ_42/40_ ratio correctly identified 14 of the 17 PET^+^ cases (82.4%) and 18 of the 21 PET^−^ individuals (85.7%). Three PET^−^ patients were misclassified as PET^+^ (false positives) and 3 PET^+^ patients were misclassified as PET^−^ individuals (false negatives) (see Additional file 5).

## Discussion

Chronic activation of microglia and astrocytes as a response to misfolded and aggregated proteins contributes to disease progression and severity in AD [13]. We thus hypothesized that the presence of inflammatory markers in the CSF might reflect this microglia activation state and might yield potential candidate biomarkers. We have recently described that GPNMB is strongly upregulated in a subset of microglia cells in the APP/PS1KI [19] and 5XFAD mouse models of AD [20]. This indicated that GPNMB could be part of a distinct microglia activation state present under neurodegenerative conditions, which is further characterized by the upregulation of genes such as *TREM2*, *APOE*, or *CST7* [20]. Recent gene expression profiling data from the APP^NL-G-F^ knock-in mouse model supports this assumption, showing that activated response microglia (ARM) are composed of specialized subgroups overexpressing MHC type II and putative tissue repair genes such as *GPNMB* [36]. Single cell RNA sequencing studies in the naïve mouse brain have revealed that GPNMB is present in a cluster of microglia with amoeboid morphology designated “axonal tract-associated microglia” (ATM), together with *Spp1*, *Igf1*, *CD68*, and *Lgals3* [37], which in part have also been identified in “disease-associated microglia” (DAM) [38, 39].

Here, we assessed whether an electrochemiluminescenceimmunoassay for the measurement of GPNMB could be applied to the study of CSF samples, and whether GPNMB CSF levels have the potential to discriminate between subjects with probable AD and non-AD disease controls, in order to further support a clinical diagnosis.

We initially evaluated the specificity of a commercial GPNMB immunoassay by analyzing cell culture supernatants and cell lysates from GPNMB-deficient macrophage-like cells generated by CRISPR/Cas9. Protein levels of soluble GPNMB were measured in conditioned cell supernatants from GPNMB knock-out cell clones with the osteoactivin MSD R-Plex assay kit, yielding assay signals below the detection limit while supernatants from control cells showed robust GPNMB levels. This perfectly corresponded with the results of a qPCR analysis, which demonstrated barely detectable levels of GPNMB mRNA expression in the GPNMB knock-out cells. Having confirmed the specificity of the GPNMB immunoassay, we next investigated serial dilutions of a pooled normal human CSF control sample and 3 individual CSF patient samples to determine suitable assay conditions. No appreciable influence of potentially interfering substances (“matrix effects”) was observed. Thus, for all subsequent CSF measurements, we decided to use 10-fold diluted CSF, which is in line with the manufacturers’ recommendation for blood plasma or serum. The reproducibility of the GPNMB protein measurements was assessed by including aliquots of pooled normal human CSF on each plate for quality control and normalization. The observed coefficient of variation for these control samples between the different assay runs was lower than 7%.

Elevated CSF GPNMB levels have been suggested as a promising biomarker candidate in other neurological disorders such as amyotrophic lateral sclerosis (ALS) and neurological forms of Gaucher disease. Quantification of CSF proteins with targeted multiple reaction monitoring mass spectrometry revealed increased GPNMB levels in short-lived ALS patients [40], a finding that was recently confirmed in an independent cohort [41]. Using multiple reaction monitoring, Oeckl and colleagues identified a tryptic peptide sequence of GPNMB mapping to the extracellular domain (364–373) in the CSF of ALS patients [41]. It has been repeatedly shown that GPNMB can undergo ectodomain shedding, resulting in the secretion of the extracellular domain [42–44], which might suggest that mainly secreted GPNMB is present in the CSF. Quantitative proteomic analyses identified GPNMB as a marker of brain pathology in Gaucher disease, a recessive inherited metabolic disorder caused by defects in the glucosylceramidase gene [45, 46]. In addition, an analysis of freshly frozen post-mortem human brain samples revealed increased GPNMB levels in the substantia nigra of sporadic Parkinson’s disease (PD) patients compared to healthy control subjects [47] and transgenic overexpression of GPNMB reduced gliosis and microglial morphological changes in a 1-methyl-4-phenyl-1,2,3,6-tetrahydropyridine (MPTP)-induced mouse model of PD [48].

In a previous small-scale pilot study including 10 AD patients and 10 non-demented controls and employing a conventional ELISA kit, we observed significantly increased CSF GPNMB levels in the AD group [20]. A recent study utilizing an integrative multiple proteomic approach of cortex, CSF, and serum samples also identified GPNMB as a potential CSF AD biomarker candidate [49]. Validation with an ELISA assay confirmed an elevation of CSF GPNMB levels in AD compared to control cases, but with a very small sample size of only 7 per diagnostic group [49]. Another deep proteomic profiling analysis of CSF samples from AD and control cases also found GPNMB as consistently changed in AD CSF, but again the sample size was very small (5 control and 8 AD cases) [50]. In contrast, in our present study, we did not detect a significant difference in CSF GPNMB levels in a larger cohort of disease controls and AD samples (*p* = 0.079).

In brain samples, GPNMB seems to be localized primarily in microglial cells surrounding extracellular Aβ deposits [20, 24]. Brain amyloid PET imaging has been proven useful to support an AD diagnosis of patients that otherwise present with a high level of diagnostic uncertainty [51]. High sensitivity and specificity of amyloid-PET imaging for the detection of neuritic amyloid plaques was confirmed by histopathological data [52]. In order to verify the results from our histological studies [20], we investigated a second cohort that was dichotomized exclusively based on amyloid-PET status. While we detected a high accuracy of 84% of the CSF Aβ_42/40_ ratio to classify PET^+^ or PET^−^ negative individuals, no discrimination was achieved using CSF GPNMB levels.

ApoE is an important protein involved in cholesterol transport, and ApoE isoforms differentially affect brain clearance of Aβ peptides [53]. As seen in cohort 1, in the present study, ApoE4 alleles are much more frequently present in patients suffering from AD compared to non-AD disease controls [54]. It has further been shown that ApoE4 carriers have a greater Aβ burden as determined by PET imaging [37], which is also visible as a trend in the PET^+^ group of cohort 2 despite of the small sample size.

### Limitations

A limitation of the current study is the lack of non-demented but otherwise healthy control individuals. As healthy individuals usually do not undergo cerebrospinal puncture, only disease-control samples were available. As most of the control samples employed in this analysis were derived from patients with a psychiatric or neurological diagnosis, we cannot rule out that ongoing inflammatory changes in these cases also affect CSF GPNMB levels, thereby masking the potential diagnostic value of an inflammation-related biomarker. Moreover, future studies may address the usefulness of GPNMB as a potential biomarker in other neurological diseases such as Parkinson’s disease or ALS.

## Conclusions

Taken together, the results from this study validate the specificity of a commercial GPNMB immunoassay and demonstrate its usability in CSF samples. An important limitation of the current study is the use of disease controls with unknown inflammatory status, warranting further studies comprising cohorts with a sufficient number of healthy non-demented individuals to further assess the validity of GPNMB as a potential diagnostic biomarker of AD.

## Supplementary Information

**Additional file 1.** A pooled normal human CSF sample (Innovative Research) and three individual CSF samples were measured in different dilutions. The CSF GPNMB concentrations were back-calculated and plotted against the dilution factor for each sample.

**Additional file 2.** Histograms of the distribution of the Aβ_42_/Aβ_40_ ratios (a) and GPNMB (b) in study cohort 1. The Aβ_42/40_ ratios had been determined in a previous study [31], and study cohort 1 comprised a subset of the clinical sample investigated and reported there.

**Additional file 3.** Combined ROC analysis for GPNMB and ApoE ε4 genotype (a) and diagnostic accuracy of the CSF GPNMB levels at the maximum Youden Index for the classification of DC and AD cases (b).

**Additional file 4.** Histograms of the distribution of the Aβ_42_/Aβ_40_ ratio (a) and GPNMB (b) in study cohort 2.

**Additional file 5.** ROC curve of MSD CSF Aβ_42/40_ ratio (a) and diagnostic accuracy of the CSF Aβ_42/40_ ratio at the maximum Youden Index for the classification of amyloid-PET^+^ vs. amyloid-PET^−^ cases (b).

## Data Availability

The datasets used and/or analyzed in the present study are available from the corresponding author on reasonable request. We acknowledge support by the Open Access Publication Funds of the Göttingen University.

## Abbreviations

Aβ: Amyloid-β
AD: Alzheimer’s disease
ALS: Amyotrophic lateral sclerosis
APOE: Apolipoprotein E
APP: Amyloid precursor protein
cDNA: Complementary DNA
CSF: Cerebrospinal fluid
EGFP: Enhanced green fluorescent protein
GPNMB: Glycoprotein non-metastatic melanoma protein B
LOD: Level of detection
PCR: Polymerase chain reaction
PET: Positron emission tomography
PMA: phorbol myristate acetate
pTau: Phosphorylated Tau protein
TREM2: Triggering receptor expressed on myeloid cells 2
ROC: Receiver operating characteristics
SUVR: Standard uptake value ratio
