# Supplementary material for: Evaluation of cerebrospinal fluid glycoprotein NMB (GPNMB) as a potential biomarker for Alzheimer’s disease
Source: Alzheimers Res Ther. 2021 May 4;13:94. doi: 10.1186/s13195-021-00828-1 (PMC8097817; doi:10.1186/s13195-021-00828-1)
Supplement: Supplementary file 1 — Additional file 1. A pooled normal human CSF sample (Innovative Research) and three individual CSF samples were measured in different dilutions. The CSF GPNMB concentrations were back-calculated and plotted against the dilution factor for each sample. [file 13195_2021_828_MOESM1_ESM.pdf]

Aichholzer et al., Evaluation of cerebrospinal fluid glycoprotein NMB (GPNMB) as a potential biomarker for Alzheimer's disease

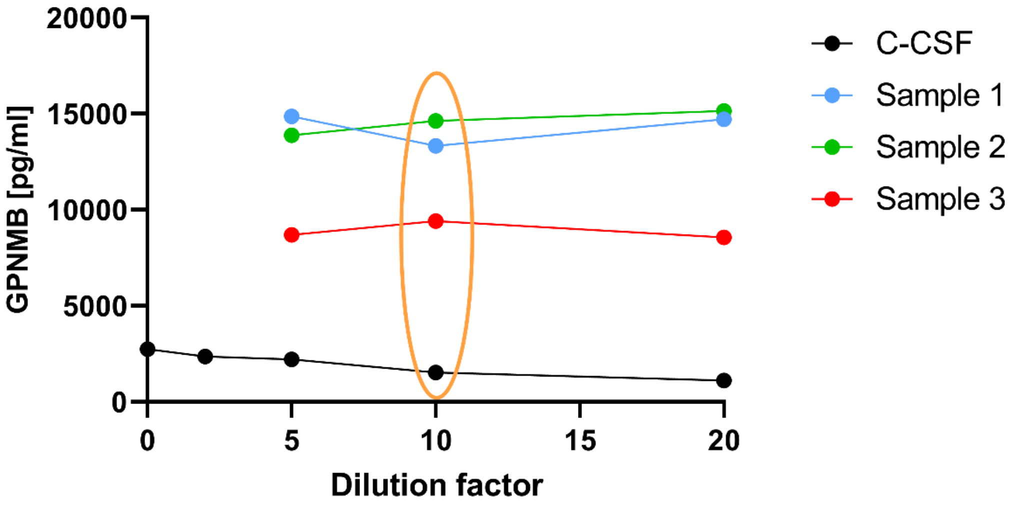

Additional file 1: A pooled normal human CSF sample (Innovative Research) and three individual CSF samples were measured in different dilutions. The CSF GPNMB concentrations were back-calculated and plotted against the dilution factor for each sample.
