# Supplementary material for: Evaluation of cerebrospinal fluid glycoprotein NMB (GPNMB) as a potential biomarker for Alzheimer’s disease
Source: Alzheimers Res Ther. 2021 May 4;13:94. doi: 10.1186/s13195-021-00828-1 (PMC8097817; doi:10.1186/s13195-021-00828-1)
Supplement: Supplementary file 2 — Additional file 2. Histograms of the distribution of the Aβ42/Aβ40 ratios (a) and GPNMB (b) in study cohort 1. The Aβ42/40 ratios had been determined in a previous study [31], and study cohort 1 comprised a subset of the clinical sample investigated and reported there. [file 13195_2021_828_MOESM2_ESM.pdf]

Aichholzer et al., Evaluation of cerebrospinal fluid glycoprotein NMB (GPNMB) as a potential biomarker for Alzheimer's disease

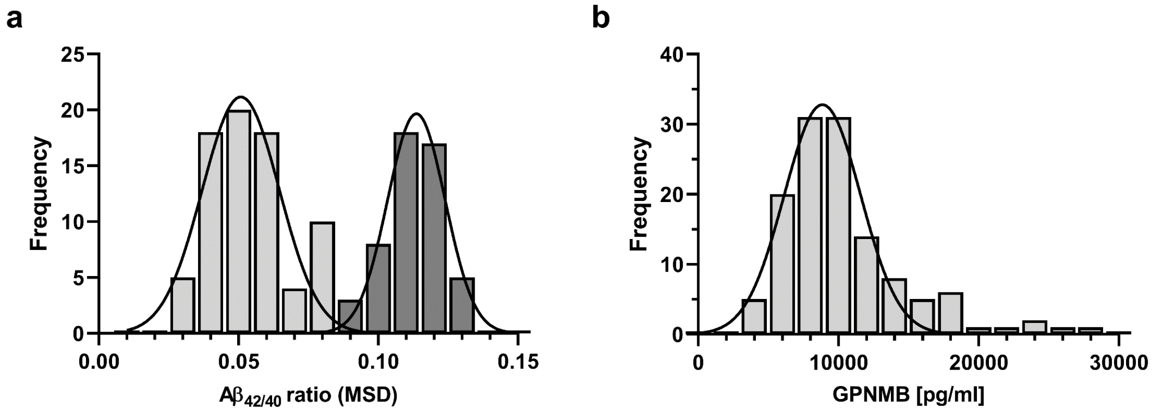

Additional file 2: Histograms of the distribution of the $A\beta_{42}/A\beta_{40}$ ratios (**a**) and GPNMB (**b**) in study cohort 1. The $A\beta_{42/40}$ ratios had been determined in a previous study (Klafki et al., J Alzheimers Dis 2016), and study cohort 1 comprised a subset of the clinical sample investigated and reported there.
