# Supplementary material for: Evaluation of cerebrospinal fluid glycoprotein NMB (GPNMB) as a potential biomarker for Alzheimer’s disease
Source: Alzheimers Res Ther. 2021 May 4;13:94. doi: 10.1186/s13195-021-00828-1 (PMC8097817; doi:10.1186/s13195-021-00828-1)
Supplement: Supplementary file 3 — Additional file 3. Combined ROC analysis for GPNMB and ApoE ε4 genotype (a) and diagnostic accuracy of the CSF GPNMB levels at the maximum Youden Index for the classification of DC and AD cases (b). [file 13195_2021_828_MOESM3_ESM.pdf]

Aichholzer et al., Evaluation of cerebrospinal fluid glycoprotein NMB (GPNMB) as a potential biomarker for Alzheimer's disease

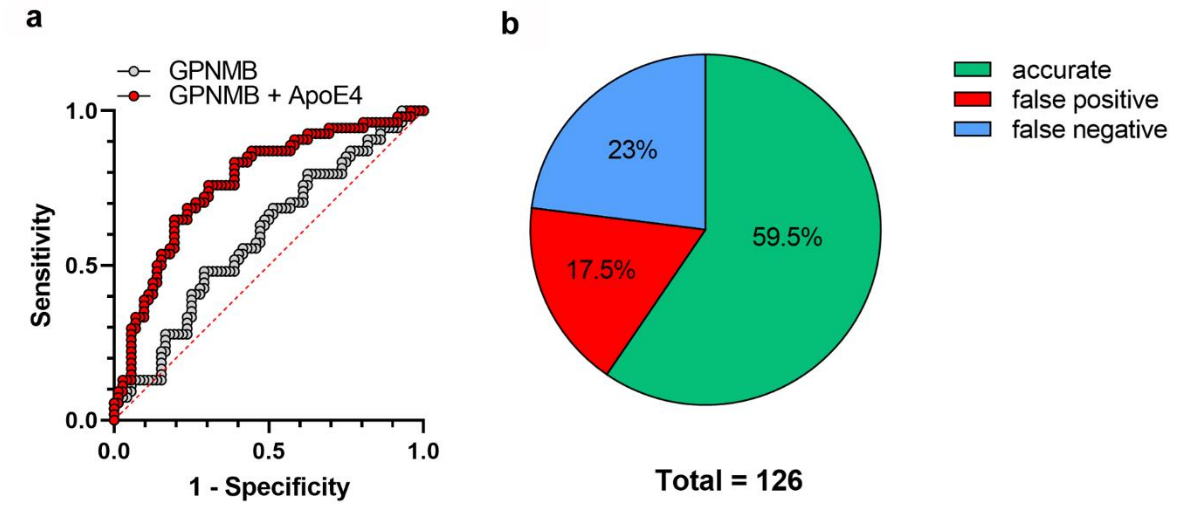

Additional file 3: Combined ROC analysis for GPNMB and ApoE ε4 genotype (**a**) and diagnostic accuracy of the CSF GPNMB levels at the maximum Youden Index for the classification of DC and AD cases (**b**).
