# Supplementary material for: Evaluation of cerebrospinal fluid glycoprotein NMB (GPNMB) as a potential biomarker for Alzheimer’s disease
Source: Alzheimers Res Ther. 2021 May 4;13:94. doi: 10.1186/s13195-021-00828-1 (PMC8097817; doi:10.1186/s13195-021-00828-1)
Supplement: Supplementary file 4 — Additional file 4. Histograms of the distribution of the Aβ42/Aβ40 ratio (a) and GPNMB (b) in study cohort 2. [file 13195_2021_828_MOESM4_ESM.pdf]

Aichholzer et al., Evaluation of cerebrospinal fluid glycoprotein NMB (GPNMB) as a potential biomarker for Alzheimer's disease

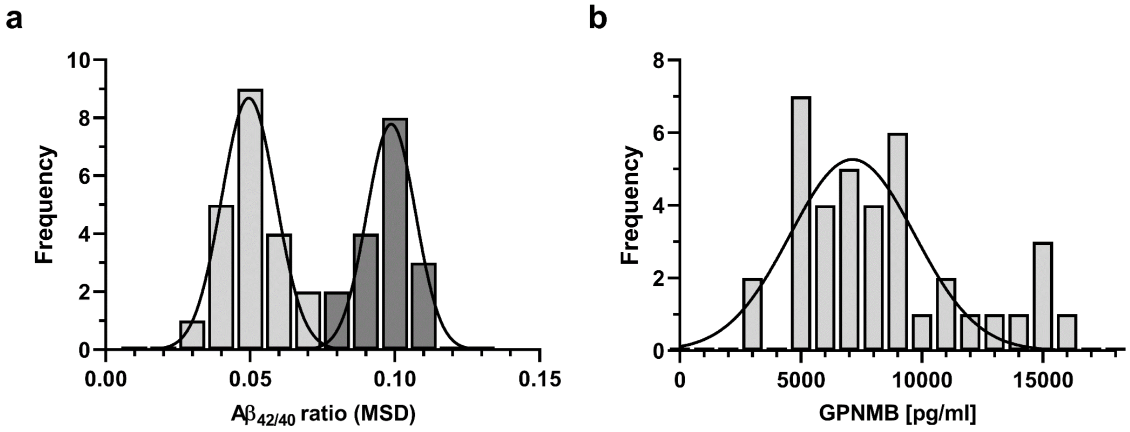

Additional file. 4: Histograms of the distribution of the $A\beta_{42}/A\beta_{40}$ ratio (**a**) and GPNMB (**b**) in study cohort 2.
