# Supplementary material for: Evaluation of cerebrospinal fluid glycoprotein NMB (GPNMB) as a potential biomarker for Alzheimer’s disease
Source: Alzheimers Res Ther. 2021 May 4;13:94. doi: 10.1186/s13195-021-00828-1 (PMC8097817; doi:10.1186/s13195-021-00828-1)
Supplement: Supplementary file 5 — Additional file 5. ROC curve of MSD CSF Aβ42/40 ratio (a) and diagnostic accuracy of the CSF Aβ42/40 ratio at the maximum Youden Index for the classification of amyloid-PET+ vs. amyloid-PET− cases (b). [file 13195_2021_828_MOESM5_ESM.pdf]

Aichholzer et al., Evaluation of cerebrospinal fluid glycoprotein NMB (GPNMB) as a potential biomarker for Alzheimer's disease

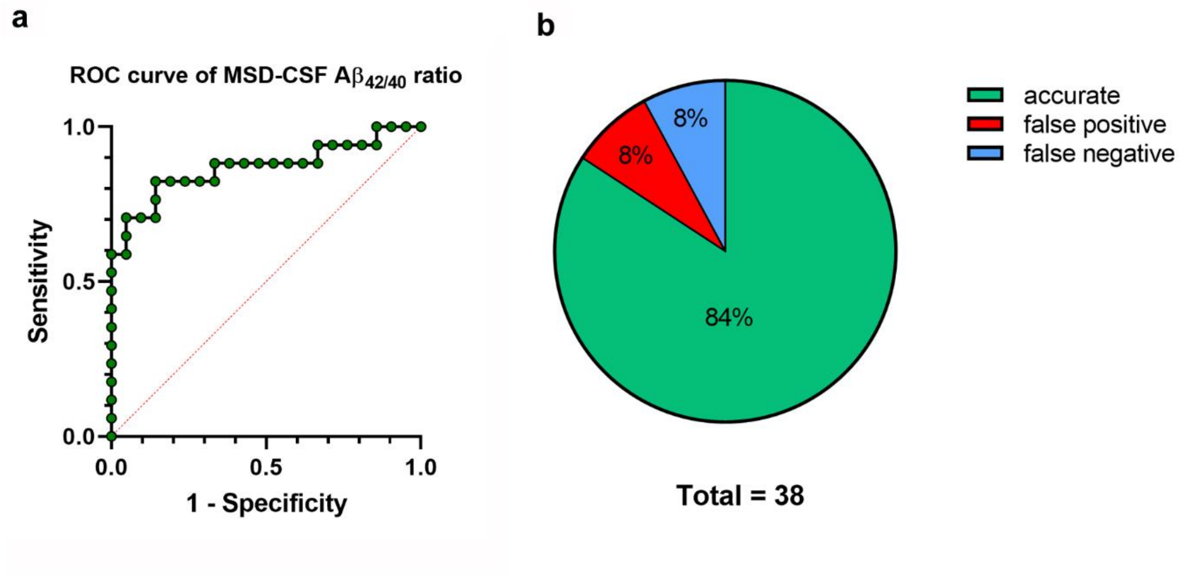

Additional file 5: ROC curve of MSD CSF $A\beta_{42/40}$ ratio (**a**) and diagnostic accuracy of the CSF $A\beta_{42/40}$ ratio at the maximum Youden Index for the classification of amyloid-PET^{+} vs. amyloid-PET^{-} cases (**b**).
